# Identification of metastasis driver genes by massive parallel sequencing of successive steps of breast cancer progression

**DOI:** 10.1371/journal.pone.0189887

**Published:** 2018-01-02

**Authors:** Anne Bruun Krøigård, Martin Jakob Larsen, Anne-Vibeke Lænkholm, Ann S. Knoop, Jeanette Dupont Jensen, Martin Bak, Jan Mollenhauer, Mads Thomassen, Torben A. Kruse

**Affiliations:** 1 Department of Clinical Genetics, Odense University Hospital, Odense, Denmark; 2 Human Genetics, Institute of Clinical Research, University of Southern Denmark, Odense, Denmark; 3 Department of Pathology, Slagelse Hospital, Slagelse, Denmark; 4 Department of Oncology, Rigshospitalet, Copenhagen, Denmark; 5 Department of Oncology, Odense University Hospital, Odense, Denmark; 6 Department of Pathology, Odense University Hospital, Odense, Denmark; 7 Lundbeckfonden Center of Excellence NanoCAN, University of Southern Denmark, Odense, Denmark; 8 Molecular Oncology Group, Institute of Molecular Medicine, University of Southern Denmark, Odense, Denmark; University of Alabama at Birmingham, UNITED STATES

## Abstract

Cancer results from alterations at essential genomic sites and is characterized by uncontrolled cell proliferation, invasion and metastasis. Identification of driver genes of metastatic progression is essential, as metastases, not primary tumors, are fatal. To gain insight into the mutational concordance between different steps of malignant progression we performed exome sequencing and validation with targeted deep sequencing of successive steps of malignant progression from pre-invasive stages to asynchronous distant metastases in six breast cancer patients. Using the ratio of non-synonymous to synonymous mutations, a surprisingly large number of cancer driver genes, ranging between 3 and 145, were estimated to confer a selective advantage in the studied primary tumors. We report a substantial amount of metastasis specific mutations and a number of novel putative metastasis driver genes. Most notable are the *DCC*, *ABCA13*, *TIAM2*, *CREBBP*, *BCL6B* and *ZNF185* genes, mainly mutated exclusively in metastases and highly likely driver genes of metastatic progression. We find different genes and pathways to be affected at different steps of malignant progression. The Adherens junction pathway is affected in four of the six studied patients and this pathway most likely plays a vital role in the metastatic process.

## Introduction

Cancer evolves through the stochastic, cumulative acquisition of driver mutations disrupting key pathways leading to the hallmarks of cancer [1]. A cancer driver mutation confers a selective advantage, while passenger mutations are coexisting mutations in the successfully expanding clones [2]. The cancer genome evolves dynamically influenced by the generation of additional mutations and selective forces acting on cancer clones, the latter being time and site dependent. The term *oncogene addiction* [3] describes the cancer cell dependence of particular driver genes for maintenance of the malignant phenotype and provides the rationale for targeted therapy. One of the major challenges in cancer genetics is to identify cancer driver genes.

Mutations in the coding region can be divided into synonymous, also known as silent mutations, and non-synonymous mutations. Typically, nucleotide substitutions in the third codon position are silent, whereas substitutions in the first and second codon positions result in an amino acid change. The ratio of non-synonymous to synonymous mutations (NS:S ratio) has been used as a reliable indicator of selection. Two factors influence the NS:S ratio, including the rate of creation and the selective forces acting on them. In the absence of selection, non-synonymous and synonymous mutations are equally likely to persist [4] and thus the NS:S ratio can indicate whether or not selection is occurring.

The metastatic process is highly complex and not yet fully understood. The main bottleneck for metastasis formation is believed to be colonization at the distant site [5]. A solid tumor is suggested to infiltrate into the circulatory system one million cancer cells per day [6] and tumor cells are found to disseminate systemically even from pre-invasive tissue [7]. Thus, additional genetic, epigenetic or host response events are needed in order to allow a disseminated tumor cell to create a metastatic lesion. Identification of specific driver genes of the metastatic process is to a large degree limited to the yet relatively few identified metastasis suppressor genes [8]. A reduced expression of a metastasis suppressor gene does not provide a selective advantage in the primary tumor, but plays a major role in the metastatic process [9]. Based on their level of participation in different steps of the metastatic process different classes of metastasis genes have been suggested: *metastasis initiation genes*, *metastasis progression genes* and *metastasis virulence genes* [10]. In addition to acquiring abilities like detachment, motility, invasion, intravasation, survival in the circulation and adaptation to new environment the malignant cell must be able to evade immune surveillance. Microenvironmental factors like acidity and hypoxia also provide selective forces upon the cancer clones [2]. Thus, the driving capacity of mutations is site dependent, inducing genetic disparity between a primary tumor and its metastases. Therapy-induced eradication of the dominant, chemotherapy- and anti-hormonal therapy sensitive clones serves to increase the selective pressure within the malignant cell population, leading to expansion of therapy-resistant clones. Hence, the location of a recurrence and treatment influences molding of the cancer genome at the distant site. Due to increasing genomic instability and stochastic events [9] cancer genome evolution must be expected to continue also in disseminated tumor cells after removal of the primary tumor.

In a recent study, including only two breast cancer patients, gene expression signatures, DNA copy number patterns and somatic mutation patterns were found to be highly similar across primary tumors and matched metastases [11]. In another study, including 11 patients, a high concordance of chromosomal rearrangements was found between primary tumors and matched metastases [12].

Mutational discordances between a primary tumor and its metastases may identify new driver genes of metastatic progression and provide insight into the biology underlying metastatic progression. In our study, we have used exome and deep targeted sequencing of pre-invasive stages, primary tumors, synchronous axillary lymph node (ALN) metastases and asynchronous distant metastases from six breast cancer patients to identify putative novel driver genes of metastatic progression and to identify pathways involved in metastasis.

## Materials and methods

### Patient material

The study includes successive tumor samples from six breast cancer patients with estrogen receptor (ER) positive invasive ductal carcinoma. Table 1 displays clinical information of the patients. All patients had ALN metastases at the time of diagnosis and primary tumors from all patients and synchronous ALN metastases from five of the patients were secured during primary surgery and stored at -80°C until sample preparation. In three cases, also pre-invasive stages, Ductal Carcinoma in Situ (DCIS) were secured during primary surgery. In one case, Patient ID (PTID) 8, we had access to two different regions of DCIS and in one case, two different regions of primary tumor (PTID 4). In spite of adjuvant therapy, four of the patients experienced recurrence of the disease, with a median relapse time of 3.08 years, and asynchronous metastases were biopsied from bone, lymph node and in two cases liver, respectively. Haematoxylin-eosin sections of all tissue samples were reviewed by a certified pathologist, ensuring the diagnosis and a content of malignant cells of 50% at minimum. A start amount of 20–30 mg fresh frozen tissue (asynchronous metastasis 5 mg) was used for the purification process. Tissue disruption and homogenization was performed using TissueLyser (Qiagen) and purification of DNA was performed using AllPrep DNA/RNA Mini Kit (Qiagen). Matched normal tissue and the primary tumor of PT ID 8 were stored as formalin-fixed paraffin-embedded (FFPE) tissue. The FFPE blocks were cut in 30–40 sections of 10 μm and DNA extracted using AS1000 Maxwell 16 (Promega, USA).

**Table 1:** Patient characteristics.

Pt ID	Age	Type	Primary size	ER	PR	HER 2	MG	# pos LN	Relapse time	Treatment
4	72 years	IDC	14 mm	pos	neg	A	II	1/10 LN	1.82 years	Surgery, adjuvant Letrozole.
8	58 years	IDC	50 mm	pos	pos	N	II	15/15 LN	4.05 years	5 series of neo-adj. CEF, surgery, 4 series of Taxotere/Gemcitabine. Tamoxifen 2.5 years, then Arimidex, radiation therapy
11	46 years	IDC	25 mm	pos	pos	N	III	1/15 LN	2.57 years	Surgery, adjuvant 7 series of CEF, Tamoxifen, radiation therapy.
15	66 years	IDC	17 mm + 15 mm = multifokal	pos	pos	N	III	17/18 LN	3.90 years	Surgery, adjuvant Letrozole, radiation therapy.
46	79 years	IDC	10 mm and diffusely spread 110 mm	pos	neg	N	III	5/20 LN		Neo-adjuvant Letrozole, surgery.
123	67 years	IDC	23 mm	pos	neg	A	II	5/11 LN		Surgery.

IDC: Invasive ductal carcinoma. ER: Estrogen receptor status. MG: Malignancy grade. PR: Progesterone receptor status. N: normal. A: amplified. CEF: Cyclofosfamid, Epirubicin, 5- Flouracil. LN: lymph nodes.

Additional analyses of the data have been described in [13] and [14].

The patients provided written informed consent to participate in the study and for the data to be published. The study was approved by the Ethical Committee of Region Syddanmark and notified to the Danish Data Protection Agency.

### Library construction and exome sequencing

One microgram of genomic DNA from each sample was randomly fragmented by focused acoustic shearing (Covaris inc.) according to Illumina’s protocol. The fragment length was measured by Bioanalyzer (Agilent Technologies 2100), confirming a fragment length of 150–300 bp. Exome enrichment was performed with Illumina’s TruSeq DNA Sample Preparation. Paired end sequencing of 2 x 100 bases was performed on the Illumina HiSeq 1500 platform. FASTQ files were aligned to the human reference genome GRCh37 (feb.2009) using the Novoalign v. 3 algorithm (www.novocraft.com) at default parameters. Removal of duplicate reads, recalibration and local realignment around indels were performed using Best Practices pipeline v. 2.7 [15]. The result was a mean coverage rate in the exome region of 65–155 x in the tumor samples and 11–148 x in the matched normal samples (S1 Table).

### Detection of putative somatic mutations for deep sequencing

On the exome sequencing data, somatic variant calling was performed using nine publicly available somatic variant callers: EB Call [16], Mutect [15], Seurat [17], Shimmer [18], Indelocator (http://www.broadinstitute.org/cancer/cga/indelocator), Somatic Sniper [17], Strelka [19], Varscan 2 [20] and Virmid [21]. The union of putative somatic mutations, except positions in intronic, intergenic, downstream and non-coding RNA intronic areas, reported by the nine somatic variant callers was used to select chromosomal candidate regions for targeted deep sequencing.

### Validation with targeted deep sequencing

Target enrichment was performed using SureSelect DNA enrichment methodology (Agilent). A custom SureSelect enrichment kit was designed using the Agilent SureDesign application. Library construction and SureSelect enrichment were performed according to manufacturer’s protocol and sequenced on the Illumina HiSeq 1500 platform with paired end sequencing 2 x 100 bases. Deep sequencing resulted in a mean coverage of 221–628 x of the targeted positions (S2 Table). Alignment and data preprocessing were performed as described previously. Variant calling were performed using Varscan 2 [20] version 2.3.6 (multisample setting). For each patient the following criteria were used: normal sample B Allele Frequency (BAF) less than 0.02, all samples should have a read depth of min. 50 x and BAF in one of the tumor samples should be 0.05 at minimum. For positions meeting those criteria, a mutation found with a BAF of 0.025 at minimum was included in other tumor samples if read depth exceeded 200 x. The variants were annotated with Annovar [22] and only exonic and splicing variants were included for further analysis. Known SNPs with a population allele frequency > 1% were excluded.

Subsequently, all identified somatic mutations within the coding region were manually curated, by visual inspection of the BAM files to remove false positive calls. Variants located in a repetitive area and variants with many adjacent variants were excluded, as they most likely result from systematic misalignment. Furthermore, unrelated BAM files were compared to the patient BAM files in order to identify error prone regions.

### Pathway analysis of genes involved in different steps of malignant progression

The non-synonymous and splice site mutations were divided into three categories:

1. Mutations found exclusively in DCIS and primary tumors.
2. Mutations shared between primary tumors and metastases.
3. Mutations found exclusively in metastases.

Pathway analysis for overrepresentation of genes in KEGG gene sets (http://www.genome.jp/kegg/) were computed for each category of genes using the online tool Molecular Signatures Database by Broad Institute [23,24].

### Identification of putative novel driver genes of metastatic progression

In order to prioritize among the many missense mutations, the iCAGES software tool (http://icages.usc.edu) was used in order to facilitate the distinction between driver and passenger mutations. The iCAGES prioritization of putative cancer driver variants uses a radial Support Vector Machine (SVM) based on nine functional prediction tools (SIFT, PolyPhen-2, GERP++, FATHMM, Mutation Taster, Mutation Assessor, Siphy, PhyloP, LRT) which is trained on somatic non-synonymous SNVs from the Catalogue Of Somatic Mutations In Cancer (http://cancer.sanger.ac.uk/cancergenome/projects/cosmic) and the Uniprot databases (http://www.uniprot.org). The resulting radial SVM predicted score evaluates the cancer driver potential for each particular mutation. Additionally the iCAGES tool incorporates a Phenolyzer score evaluating the genetic-phenotypic association based on previous database knowledge (http://phenolyzer.usc.edu). A total weighed score, iCAGES score, ranks each mutation according to cancer driver potential.

Putative novel drivers of malignant progression were selected by the following criteria: Classified as a cancer driver gene by the iCAGES software or affected by a frameshift, stopgain or splicing mutation.

## Results

### Varying mutational concordance among the analyzed patients

**Fig 1:**
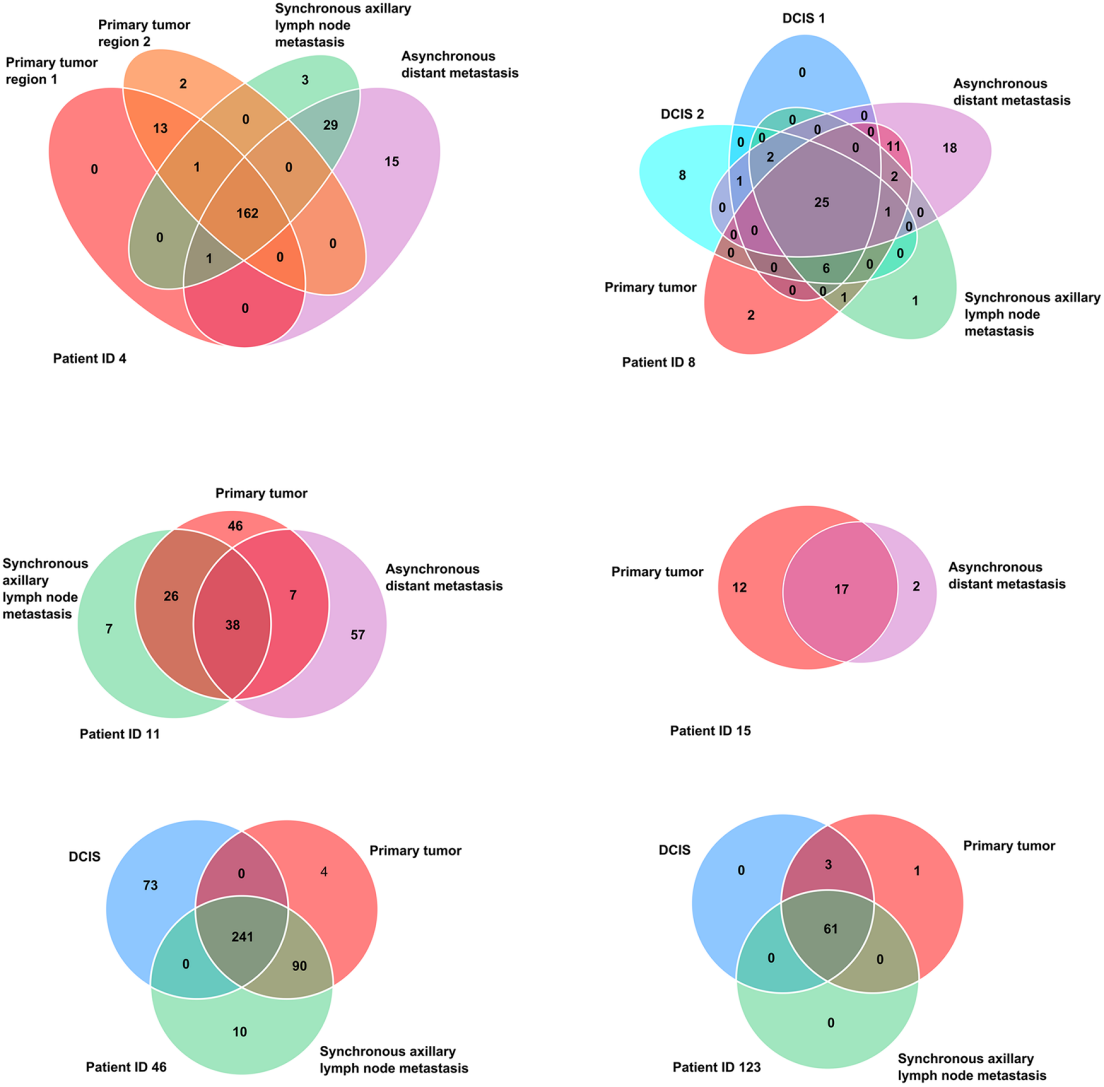
Genomic concordance. Venn diagrams depicting the genomic concordance of non-synonymous and splice site mutations between different steps of progression in the six studied patients.

A large variation in the number of mutations in the coding region of the studied patients was found. A total of 31–418 non-synonymous and splicing mutations and 13–113 synonymous mutations were identified by exome sequencing and validated by targeted deep sequencing in the six patients included in the study. The complete lists of validated somatic mutations in the coding region of the six studied patients are available in S3–S8 Tables. Venn diagrams displaying the mutational concordance of non-synonymous and splice site mutations between different steps of malignant progression are shown in Fig 1. The patients display varying degree of genetic concordance between different steps of progression. The majority of mutations are shared between primary tumors and metastases, however, a significant number of mutations are exclusive to the metastases of the studied patients.

### The ratio of non-synonymous and synonymous mutations indicates a considerable number of driver genes

The number of non-synonymous (NS) and synonymous (S) mutations identified in the primary tumors of each patient are shown in Table 2. NS:S ratios in four of the six studied primary tumors are significantly higher than the NS:S ratio of 2:1 predicted for non-selected passenger mutations. For the remaining two primary tumors, the numbers are probably too small to reach significance. The number of cancer driver mutations, conferring a selective advantage to the malignant cells, can be calculated as the number of non-synonymous mutations exceeding the expected ratio of 2:1. With reservations for our assumptions, the number of driver genes in the primary tumors included in our study varies between 3 and 145 (S9 Table). The NS:S ratios for ALN metastases specific mutations collectively reaches statistical significance (Table 3), while the mutations specific to the asynchronous metastases do not reach the normally accepted statistical significance level of 5%.

**Table 2:** Non-synonymous and synonymous mutations present in the primary tumors.

	PT ID 4	PT ID 8	PT ID 11	PT ID 15	PT ID 46	PT ID 123
Non-synonymous	177	48	117	29	335	65
Synonymous	47	16	42	13	95	21
Ratio	3.76	3.0	2.78	2.23	3.52	3.09
P-value	0.00003	0.097	0.036	0.44	2.08 E 10^−7^	0.047

P-values are calculated by one-tailed binomial test.

**Table 3:** Non-synonymous and synonymous mutations specific for mutations in ALN metastases and asynchronous distant metastases, respectively, collectively for the six studied patients.

	ALN metastasis specific mutations	Asynchronous distant mutation specific mutations
Non-synonymous	50	92
Synonymous	15	35
Ratio	3.33	2.62
P-value	0.049	0.097

P-values are calculated by one-tailed binomial test.

### Recurrently mutated genes in metastatic progression

A number of genes are recurrently mutated within individual patient samples and across the six studied patients as seen in Tables 4 and 5, respectively. Some of the genes are already established as cancer related genes as they are present in the Catalogue Of Somatic Mutations In Cancer (COSMIC) Cancer Gene Census list (http://cancer.sanger.ac.uk/cancergenome/projects/census/) while the remaining genes may include novel cancer driving genes.

**Table 4:** Recurrently mutated genes within individual patient samples.

	Cosmic CGC	Patient ID 4				Patient ID 8					Patient ID 11			Patient ID 15		Patient ID 46			Patient ID 123		
Gene		PT 1	PT 2	ALNM	DM	DCIS 1	DCIS 2	PT	ALNM	DM	PT	ALNM	DM	PT	DM	DCIS	PT	ALNM	DCIS	PT	ALNM
**MUC4**											6x	6x	x								
**FREM2**		3x	3x																		
**BRCA2**	yes	2x	2x	2x	2x																
**TTN**																x	x	2x			
**USHBP1**													3x								
**WAC**																3x	3x	3x			
**XIRP2**													3x								
**AKAP9**	yes															2x	x	x			
**ARHGAP21**		2x	2x	2x	2x																
**ARMC5**		2x	2x	2x	2x																
**BRD2**																x	2x	2x			
**C1orf63**		2x	2x	2x	2x																
**CAMK2A**		2x	2x	2x	2x																
**F5**		2x	2x	2x	2x																
**FCGBP**																x		2x			
**IL6ST**	yes										2x	x	x								
**KIAA1731**											2x	2x	2x								
**LRP1**																2x	2x	2x			
**METTL22**																2x	2x	2x			
**PCDHB16**											2x		2x								
**PLAT**																2x	2x	2x			
**PTPRK**	yes															2x	2x	2x			
**RIC8A**																2x	2x	2x			
**RUNX1**	yes															2x	2x	2x			
**TFRC**	yes															x	2x	2x			
**TIAM2**																		2x			
**UGGT2**																x2	x2	x2			
**ZNF469**											2x										
**ABCA13**																			3x	3x	3x

Only non-synonymous and splice site mutations are included. Cosmic CGC: Included in the Cosmic Cancer Gene Census. x: one mutation. 2x: two mutations. 3x: three mutations. 6x: six mutations. DCIS: ductal carcinoma in situ. DM: asynchronous distant metastasis. PT: patient. ALNM: axillary lymph node metastasis.

**Table 5:** Recurrently mutated genes across patients.

	Cosmic CGC	Patient ID 4				Patient ID 8					Patient ID 11			Patient ID 15		Patient ID 46			Patient ID 123		
Gene		PT 1	PT 2	ALNM	DM	DCIS 1	DCIS 2	PT	ALNM	DM	PT	ALNM	DM	PT	DM	DCIS	PT	ALNM	DCIS	PT	ALNM
**TP53**	yes					x	x	x	x	x	x	x	x			x	x	x			
**KIAA1033**						x	x	x	x	x						x	x	x	x	x	x
**TNXB**											x	x				x	x	x	x	x	x
**WDFY4**		x	x	x	x		x	x	x	x											
**PCDHGA1**		x	x	x	x											x	x	x			
**MAN2C1**		x	x	x	x												x	x			
**MYO3A**		x	x	x	x											x	x	x			
**RUNX1**	yes	x	x	x	x											2x	2x	2x			
**BRCA2**	yes	2x	2x	2x	2x											x	x	x			
**YY1AP1**		x	x	x	x											x	x	x			
**STK4**		x	x	x	x											x	x	x			
**LRRK1**		x	x	x	x														x	x	x
**SLC41A2**		x	x	x	x												x	x			
**CDH23**		x	x	x	x								x								
**ZNF827**		x	x	x	x														x	x	
**ABCA13**				x	x														3x	3x	3x
**LTN1**				x	x														x	x	x
**UNC80**				x	x												x	x			
**PIK3CA**	yes										x	x	x			x	x	x			
**APLF**											x	x							x	x	x
**BAI3**											x					x	x	x			
**C4orf21**											x		x						x	x	x
**DCC**															x			x			
**DNAH11**														x	x	x	x	x			
**DYNC2H1**											x	x	x			x	x	x			
**KLHL32**											x	x	x			x					
**NXNL2**											x	x	x						x	x	x
**PTPN14**											x	x	x			x	x	x			
**ROS1**	yes										x					x	x	x			
**SGCE**																x	x	x	x	x	x
**SON**											x	x	x			x	x	x			
**TLN2**											x	x					x	x			
**UTP20**														x			x	x			
**VWF**												x		x	x						
**FLG**													x			x	x	x			
**UBE2O**													x				x	x			

Includes only non-synonymous and splice site mutations. Cosmic CGC: Included in the Cosmic Cancer Gene Census. x: one mutation. 2x: two mutations. 3x: three mutations. DCIS: ductal carcinoma in situ. DM: asynchronous distant metastasis. PT: patient. ALNM: axillary lymph node metastasis.

### Different gene sets are mutated at different steps of malignant progression

The genes affected by non-synonymous and splice site mutations in the six studied patients were divided into three categories based on the steps of malignant progression in which they appear. The genes included in Categories 1–3 are listed in S10 Table. Pathway analysis on the three categories of genes revealed that different pathways are involved in the different steps of malignant progression. In Category 1, including 147 genes found to be mutated exclusively in pre-invasive tissue or primary tumors, the top five KEGG pathways included Homologous recombination and Mismatch repair pathways among others, as shown in Table 6.

**Table 6:** Results from overlap analyses, Category 1. Pathway analysis of genes mutated exclusively in DCIS and primary tumors, 147 in total.

Pathways	Genes in overlap	p-value	FDR q-value
**KEGG Homologous recombination (28)**	4	1.94 E -6	3.6 E -4
**KEGG Steroid hormone biosynthesis (55)**	3	7.45 E -4	6.93 E -2
**KEGG Primary bile acid biosynthesis (16)**	2	1.18 E -3	7.34 E -2
**KEGG Mismatch repair (23)**	2	2.46 E -3	1.14 E -1
**KEGG Dilated cardiomyopathy (92)**	3	3.27 E -3	1.22 E -1

(#): number of genes in pathway. FDR: false discovery rate.

**Fig 2:**
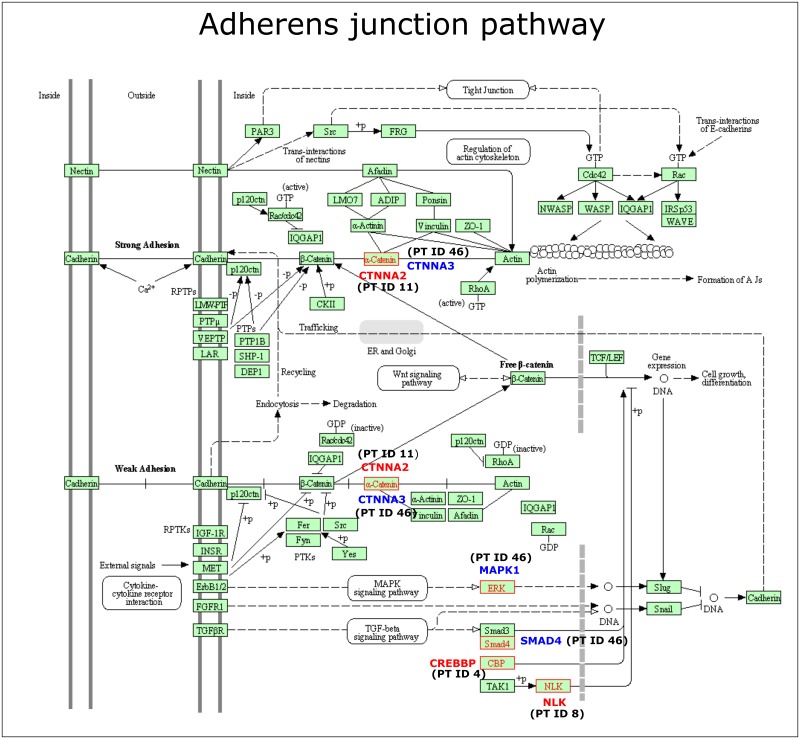
KEGG Adherens junction pathway, which is affected exclusively in the metastases of three of the studied patients (gene names in red), and affected by shared mutations between primary tumor and metastasis in one patient (gene names in blue). CTNNA2 and CTNNA3 are both encoding α-catenins. Patient IDs are in brackets.

Category 2, including 606 genes mutated in primary tumors and matched metastases, is significantly enriched for genes on various cancer related pathways, as seen in Table 7. Pathway analysis on the 129 metastasis specific genes in Category 3 reveals that the top three pathways, although not reaching statistical significance levels, are KEGG Adherens junction, KEGG Ubiquitin mediated proteolysis and KEGG Wnt signaling pathway Table 8. These pathways are likely to be key participants in the metastatic process. Three genes, *NLK* (PTID 8), *CREBBP* (PTID 4), *CTNN2A* (PTID11) are involved in the KEGG Adherens junction pathway and thus this pathway is affected in the distant metastases of three of the patients, as seen in Fig 2. Three genes, *MAPK1*, *SMAD4* and *CTNNA3*, also belonging to the Adherens junction pathway are affected by mutations in both primary tumor and metastasis of a fourth of the studied patients (PTID 46). Pathway analysis including genes mutated in both Category 2 and Category 3 reveals the Adherens junction pathway to be significantly affected (FDR q value 6.08 E -3) in the primary tumors and metastases of the studied patients.

**Table 7:** Results from overlap analyses, Category 2. Pathway analysis of genes mutated in primary tumors and corresponding metastases, 606 in total.

Pathways	Genes in overlap	p-value	FDR q-value
**KEGG Neutrophin signaling pathway (126)**	13	1.41 E -8	2.62 E -6
**KEGG Chronic meyloid leukemia (73)**	10	4.35 E -8	4.05 E -6
**KEGG Pathways in cancer (328)**	19	1.02 E -7	6.35 E -6
**KEGG Endometrial cancer (52)**	8	3.94 E -7	1.75 E -5
**KEGG Focal adhesion (201)**	14	5.51 E -7	1.75 E -5
**KEGG Steroid hormone biosynthesis (55)**	8	6.16 E -7	1.75 E -5
**KEGG MAPK signaling pathway (267)**	16	6.6 E -7	1.75 E -5
**KEGG Glioma (65)**	8	2.28 E -6	4.83 E -5
**KEGG ERBB signaling pathway (87)**	9	2.34 E -6	4.83 E -5
**KEGG Complement and coagulation cascades (69)**	8	3.6 E -6	6.7 E -5
**KEGG Non-small cell lung cancer (54)**	7	6.93 E -6	1,17 E -4
**KEGG Insulin signaling pathway (137)**	10	1.51 E -5	2.35 E -4
**KEGG Colorectal cancer (62)**	7	1.76 E -5	2.52 E -4
**KEGG Calcium signaling pathway (178)**	11	2.74 E -5	3.64 E -4
**KEGG Thyroid cancer (29)**	5	3.58 E -5	4.44 E -4
**KEGG Pancreatic cancer (70)**	7	3.91 E -5	4.55 E -4
**KEGG GnRH signaling pathway (101)**	8	6.02 E -5	6.33 E -4
**KEGG B cell receptor signaling pathway (75)**	7	6.12 E -5	6.33 E -4
**KEGG Acute myeloid leukemia (60)**	6	1.4 E -4	1.37 E -3
**KEGG Prostate cancer (89)**	7	1.81 E -4	1.69 E -3

(#): number of genes in pathway. FDR: false discovery rate.

**Table 8:** Results from overlap analyses, Category 3. Pathway analysis of genes mutated exclusively in metastases, 129 in total.

Pathways	Genes in overlap	p-value	FDR q-value
**KEGG Adherens junction (75)**	3	1.26 E -3	2.34 E -1
**KEGG Ubiquitin mediated proteolysis (138)**	3	7.03 E -3	4.43 E -1
**KEGG Wnt signaling pathway (151)**	3	8.99 E -3	4.43 E -1

(#): number of genes in pathway. FDR: false discovery rate.

### Putative novel drivers of metastatic progression can be found among genes affected by metastasis specific mutations and mutations shared between primary tumors and metastases

Exclusively in the metastases, 142 non-synonymous and splice site mutations were found. Among these, 45 mutations in 43 genes are classified as putative progression drivers as they are affected by splice site, stopgain or frameshift mutations or a missense mutation classified as an iCAGES driver mutation. The putative drivers of progression among the genes mutated exclusively in the metastases of the studied patients are listed in Table 9. Some of the genes are already present in the Cancer Gene Census list or KEGG cancer pathway (http://www.genome.jp/kegg-bin/show_pathway?hsa05200) while other genes may represent novel drivers of malignant progression. Several of the genes are likely cancer progression genes, based on literature review, while other genes have not (yet) been described in relation to cancer.

**Table 9:** Putative drivers of metastatic progression from Category 3, metastasis specific mutations.

PT ID 4	Gene	CytoBand	Mutation type	cDNA change	AA change	Category	iCAGES	ALNM BAF	DM BAF
	**LAMB3**	1q32.2	S	c.1132+2T>G				x	x
	**DUSP10**	1q41	NS	c.335A>C	p.Q112P		Yes	x	x
	**IL1RAP**	3q28	FS del	c.295_302del	p.99_101del			x	x
	**ABCA13**	7p12.3	FS del	c.7112delG	p.R2371fs			x	x
	**CREBBP**	16p13.3	NS	c.4357C>A	p.Q1453K	CGC	Yes	x	x
	**GTF2F1**	19p13.3	NS	c.808G>C	p.E270Q		Yes	x	x
	**BCL6B**	17p13.1	FS del	c.1176_1185del	p.392_395del			x	x
	**ZNF185**	Xq28	S	c.1971+2T>A				x	x
	**ACADM**	1p31.1	NS	c.271A>T	p.T91S		Yes	x	
	**ST6GALNAC3**	1p31.1	SG	c.874A>T	p.K292X			x	
	**ZAP70**	2q11.2	NS	c.920C>T	p.P307L		Yes		x
	**MYO7A**	11q13.5	NS	c.3126G>T	p.W1042C		Yes		x
	**CDH5**	16q21	NS	c.1738G>A	p.E580K		Yes		x
	**PPP2R1A**	19q13.41	NS	c.28C>A	p.L10M	CGC	Yes		x
	**WDR52**	3q13.2	FS del	c.2513delA	p.N838fs				x
	**BRD8**	5q31.2	SG	c.1921G>T	p.E641X				x
**PT ID 8**	**NCKAP5**	2q21.2	S	c.1757-2A>G					x
	**VCAN**	5q14.3	NS	c.2539G>C	p.E847Q		Yes		x
	**ARID1B**	6q25.3	NS	c.3607G>T	p.A1203S		Yes		x
	**OR1K1**	9q33.2	NS	c.160C>A	P.P54T		Yes		x
	**NLK**	17q11.2	NS	c.230C>T	p.A77V		Yes		x
	**DOCK6**	19p13.2	NS	c.2338C>A	p.L780M		Yes		x
	**GSG1L**	16p12.1	SG	c.507C>A	p.C169X				x
**PT ID 11**	**SPSB1**	1p36.22	NS	c.442C>T	p.R148W		Yes		x
	**OR2M4**	1q44	NS	c.745G>A	p.G249R		Yes		x
	**CTNNA2**	2p12	NS	c.625G>A	p.A209T	KEGG	Yes		x
	**ITPR1**	3p26.1	NS	c.7915G>A	p.E2639K		Yes		x
	**PTPN13**	4q21.3	NS	c.3015G>T	p.M1005I		Yes		x
	**GPRC6A**	6q22.1	NS	c.1637G>T	p.R546I		Yes		x
	**CSPP1**	8q13.2	FS del	c.2032delA	p.K678fs				x
	**UBE3B**	12q24.11	NS	c.3073G>C	p.E1025Q		Yes		x
	**HS3ST3A1**	17p12	NS	c.1132G>A	p.E378K		Yes		x
	**UBE2O**	17q25.1	NS	c.1661C>T	p.S554L		Yes		x
	**PTGIS**	20q13.13	NS	c.907C>T	p.P303S		Yes		x
	**PI4KA**	22q11.21	NS	c.1939G>A	p.V647M		Yes		x
	**APOL5**	22q12.3	S	c.142+1G>A					x
	**ZNRF3**	22q12.1	NS	c.1291C>G	p.H431D		Yes	x	
	**VWF**	12p13.31	NS	c.1471C>T	p.R491C		Yes	x	
**PT ID 15**	**DCC**	18q21.2	NS	c.151A>G	p.T51A	KEGG	Yes		x
**PT ID 46**	**TMPRSS11A**	4q13.2	SG	c.282G>A	p.W94X			x	
	**TIAM2**	6q25.3	NS	c.1627G>T	p.G543C		Yes	x	
	**TIAM2**	6q25.3	NS	c.1671C>A	p.F557L		Yes	x	
	**A2M**	12p13.31	NS	c.943G>A	p.E315K		Yes	x	
	**DCC**	18q21.2	NS	c.3242G>C	p.G1081A	KEGG	Yes	x	
	**FCGBP**	19q13.2	SG	c.14279C>G	p.S4760X			x	

AA change: amino acid change. ALNM: axillary lymph node metastasis. DM: asynchronous distant metastasis. BAF: B allele frequency. Del: deletion. CGS: Cosmic Cancer Gene Census. FS: frameshift. NS: non-synonymous missense. S: splicing. SG: stopgain. KEGG: KEGG cancer pathway.

Category 2, including genes affected by shared mutations between primary tumors and metastases, includes 692 mutations. Among these, 206 mutations are classified as putative metastasis progression driver genes as they are affected by splice site, stopgain or frameshift mutations or a nonsynonymous missense mutation classified as an iCAGES driver mutation. The putative driver genes of metastatic progression from Category 2 can be found in S11 Table.

## Discussion

The present study reports substantial variation in the number of mutations within the coding region and varying mutational concordance between different steps of malignant progression in the studied breast cancer patients.

A key challenge in cancer genetics is to distinguish between driver and passenger mutations. The identified high NS:S ratios in our study imply positive selection of non-synonymous mutations in the studied primary tumors and thereby indicate that a surprisingly large proportion of the identified genes have functional significance. This is in concordance with significant enrichment in Category 2 of genes involved in many cancer related pathways. The number of calculated driver mutations in the primary tumors included in our study varies greatly, ranging between 3 and 145. Other studies have attempted to estimate the number of driver mutations in solid tumors like breast cancer and suggested up to 20 driver mutations [25,26]. Supporting the notion of many driver genes, it is estimated, that each driver mutation confers only a small selective growth advantage to the cell in the order of 0.4% [27]. Progression from early tumor stages to metastatic lesions is an evolutionary process and metastatic capacity most likely results from multiple alterations, each providing a slight selective advantage at the different steps of metastasis.

We present putative novel drivers of metastatic progression from the category of genes mutated exclusively in the metastases of the studied patients (Category 3). One could argue that metastasis promoting mutations may also be present in the mutations shared between primary tumors and metastases (Category 2) as it seems likely that metastatic abilities, at least the ones required for the early steps of the metastatic process, metastasis initiation genes, are present in the cells prior to dissemination from the primary tumor. However, only clonally expanded mutations are detectable in a study like the present and therefore, a detected mutation either confers a selective advantage or is a passenger in a successful clone. Hence, for a mutation in Category 2 to be a driver of metastatic progression it should be advantageous both at the primary tumor site and in the metastatic setting. This is indeed possible for some metastatic abilities such as invasion or angiogenesis. Thus, Category 2 most likely also contains metastasis progression drivers.

Different gene sets are affected by mutations at different steps of malignant progression. In Category 1, the top five KEGG pathways included Homologous recombination and Mismatch repair. These pathways are intuitively relevant for carcinogenesis, the early stages of malignancy. Category 2 is significantly enriched for genes participating in various cancer related pathways. The Adherens junction pathway is affected by mutations exclusively in the metastases of three of the studied patients. Adherens junctions are the most common type of intercellular adhesion and limits cell movement and proliferation and are therefore likely to play a key role in the metastatic process. Metastasis-enabling mutations may, as discussed, be present already within the primary tumor. Hence, it seems relevant to include both Category 2 and Category 3 genes in a pathway analysis, revealing the Adherens junction pathway to be significantly affected in the primary tumors and metastases of the studied patients.

Recurrently mutated genes among the studied patients are highly likely cancer drivers. Three genes, *BRCA2*, *RUNX1* and *ABCA13* are affected by recurrent mutations both within patients and among patients and are previously described in relation to cancer [28–30]. The established cancer gene *PIK3CA* [31] is affected in all tumor steps of two of the studied patients. Focusing on metastases, the *ABCA13*, *DCC* and *TIAM2* genes deserve mention. The *ABCA13* gene is, in addition to mutations in all tumor steps of PTID 123, affected by a frameshift deletion exclusively in the metastases of PTID 4. An association has been found between ATP-binding cassette transporter genes like *ABCA13* and outcome in breast cancer patients, most likely due to their role in drug resistance [30]. The *DCC* gene, mutated exclusively in metastases of two of the studied patients (PTID 15 and 46), most likely plays a role in the metastatic phenotype. Studies in *Drosophila* have suggested that the *DCC* gene functions as an invasive tumor suppressor [32,33]. In a murine model of p53 deficient mammary carcinoma cells it has been reported that additional loss of *DCC* promotes metastasis formation without affecting the primary tumor phenotype [34] suggesting that the gene limits survival of disseminated tumor cells. The *TIAM2* gene is affected by two non-synonymous mutations exclusively in the metastasis of PT ID 46. *TIAM2S* expression is reported to be positively associated with metastatic phenotype of hepatocellular carcinoma cells and the gene product reported to promote growth and invasiveness [35]. *In vivo* studies revealed that *TIAM2S* expression resulted in up-regulation of N-cadherin and vimentin and redistribution of E-cadherin [35]. Thus, the *TIAM2* gene is found to function as an oncogene promoting epithelial-to-mesenchymal transition. In order for the gene to function as a progression driver in the metastatic cells in our study, the non-synonymous mutations identified in the gene should be activating mutations.

A number of genes are found to be recurrently mutated within individual patient samples. This phenomenon could be suspected to result from false positive calls. However, in this study, validation includes sequencing with a mean read depth of 465 x and called positions were manually curated, ensuring high quality of the reported findings. Whether recurrently mutated genes within a tumor specimen are situated in cis or in trans and whether they originate from the same or different cancer cell subclones cannot be established. Two inactivating mutations located at different alleles within the same cancer cells result in a total inactivation of the gene. Conversely, if two cancer subclones are affected by different mutations in the same gene this again suggests a strong selective advantage of mutations in the gene.

From the list of putative drivers of metastatic progression found only among the metastasis specific mutations the *CREBBP*, *BCL6B* and the *ZNF185* genes are the most notable. The *CREBBP* gene, mutated exclusively in the metastases of PTID 4, is an epigenetic modifier acting as a transcriptional coactivator through acetylation of histone proteins, thereby securing transcription of genes, including tumor suppressor genes and has thus been suggested as tumor suppressor [36]. The *BCL6B* gene, affected by a frameshift deletion exclusively in both metastases of PTID 4, is recently reported a novel tumor suppressor gene in hepatocellular carcinoma [37]. The *BCL6B* gene functions as a sequence-specific transcriptional repressor in the nucleus and is ubiquitously expressed in human tissue. Stable expression of the gene in hepatocellular cell lines was found to suppress cell migration and invasion and significantly reduced the incidence and severity of lung metastases in a mouse model [37]. The anti-metastatic effect of *BCL6B* was mediated by up-regulation of cell adhesion molecules like E-cadherin and down-regulation of the angiogenesis gene *VEGFA* [37]. The *ZNF185* gene, affected by a splicing mutation specifically in both metastases of PTID 4, is suggested to function as a tumor suppressor by associating with the actin-cytoskeleton and is reported to be associated with metastatic progression in colon and prostate cancer [38,39].

The studied cancer genomes most likely represent highly aggressive and treatment insensitive cases and the reported mutational spectrum is highly influenced by the selective pressures provided by treatment. Some of the mutations may play a role in therapy resistance. Mutations in the *RUNX1* gene is previously reported to correlate with aromatase inhibition resistance [40]. Our study supports this finding as, in two of the six studied patients, the *RUNX1* gene is affected by mutations in all tumor steps and the patients experienced progression of the disease in spite of treatment with the aromatase inhibitor Letrozole.

The prioritization among missense mutations for the identification of cancer driver genes is extremely challenging and no software tool can perform the distinction flawlessly. Thus, the use of the iCAGES software is just one way one could address this task.

The present study, like most other, focuses on non-synonymous mutations, as synonymous mutations are widely considered nonfunctional in cancer. However, a relatively recent study provides evidence that such silent mutations can actually act as oncogenic drivers by altering transcript splicing and therefore affect protein function [41]. In contrast to what was previously known, it is found that natural selection also acts on synonymous sites as it was found that oncogenes, in addition to being enriched for activating missense mutations, were also enriched for synonymous mutations compared to non-cancer genes [41]. A strong association was found between synonymous mutations and differential exon usage profiles in the most recurrently mutated oncogenes. Generally, tumor suppressor genes did not display the same phenomenon, except *TP53* which also had recurrent synonymous mutations that, in contrast to those in oncogenes, were adjacent to splice sites and inactivate them [41]. The study found synonymous mutations to be nonrandomly distributed across the cancer genome and that they preferentially targeted evolutionary conserved sites. Hence, synonymous mutations might confer a selective advantage to the malignant cells. In our study, we do not find evidence of selective forces acting on synonymous mutations, however, this does not preclude that a few of the identified synonymous mutations actually do function as a progression driver.

In summary, we report substantial mutational discordance between different steps of malignant progression in the studied breast cancer patients. Putative novel drivers of malignant progression are reported among the genes mutated exclusively in the metastases and among the genes shared between the primary tumors and metastases. Most notable are the *DCC*, *TIAM2*, *CREBBP*, *BCL6B* and the *ZNF185* genes, mutated exclusively in the metastases of the studied patients and highly likely driver genes of metastatic progression. High NS:S ratios reveal positive selection of non-synonymous mutations and that more than a few driver genes confer a selective advantage in the studied primary tumors. Different genes and pathways are involved at different stages of breast cancer. The Adherens junction pathway is affected in four of the six studied patients and this pathway most likely plays a vital role in the metastatic process. The considerable amount of additional mutations in the asynchronous metastases in several of the studied patients stresses the importance of molecular profiling of metastatic tissue at recurrence of breast cancer in order to provide the optimal basis for personalized medicine.

## Supporting information

S1 Table Exome sequencing data. (XLSX)

S2 Table Mean coverage of validated positions. (XLSX)

S3 Table PT ID 4 validated positions in the coding region. (XLSX)

S4 Table PT ID 8 validated positions in the coding region. (XLSX)

S5 Table PT ID 11 validated positions in the coding region. (XLSX)

S6 Table PT ID 15 validated positions in the coding region. (XLSX)

S7 Table PT ID 46 validated positions in the coding region. (XLSX)

S8 Table PT ID 123 validated positions in the coding region. (XLSX)

S9 Table Calculations of number of driver mutations within the studied primary tumors. (XLSX)

S10 Table Genes included in Categories 1, 2 and 3, divided into the patients in which they are mutated. (XLSX)

S11 Table Putative driver genes of malignant progression from Category 2, mutations shared between primary tumors and metastases. Divided into the patients in which they are mutated.
(XLSX)
